# Effect of Gamma Irradiation on the PLA-Based Blends and Biocomposites Containing Rosemary Ethanolic Extract and Chitosan

**DOI:** 10.3390/polym14071398

**Published:** 2022-03-29

**Authors:** Cornelia Vasile, Daniela Pamfil, Traian Zaharescu, Raluca-Petronela Dumitriu, Gina Mihaela Pricope, Maria Râpă, Gabriel Vasilievici

**Affiliations:** 1 Physical Chemisytry of Polymers Department, “Petru Poni” Institute of Macromolecular Chemistry (PPIMC), 700487 Iasi, Romania; rdumi@icmpp.ro; 2 National Institute for Electrical Engineering (INCDIE ICPE CA), 030138 Bucharest, Romania; traian_zaharescu@yahoo.com; 3 Veterinary and Food Safety Laboratory, Food Safety Department, 700489 Iasi, Romania; ginacornelia@yahoo.com; 4 Faculty of Materials Science and Engineering, Politehnica University of Bucharest (UPB), 060042 Bucharest, Romania; rapa_m2002@yahoo.com or; 5 National Institute for Research & Development in Chemistry and Petrochemistry (INCDCP-ICECHIM), 060021 Bucharest, Romania; gvasilievici@icechim.ro

**Keywords:** poly(lactic acid), chitosan, rosemary extract, blends, biocomposite, gamma irradiation

## Abstract

The irradiation of polymeric materials with ionizing radiation (γ-rays, X-rays, accelerated electrons, ion beams, etc.) may lead to disproportion, hydrogen abstraction, arrangements, degradation, and/or the formation of new bonds. The purpose of this paper is to evaluate the effect of gamma irradiation on some new poly(lactic acid) (PLA)-based blends and biocomposites, which is crucial when they are used for food packaging or medical purposes. The polymeric blends and biocomposites based on PLA and rosemary ethanolic extract (R) and poly(ethylene glycol) (PEG) (20 wt%) plasticized PLA, chitosan (CS) (3–6 wt%) and R (0.5 wt%) biocomposites were subjected to gamma irradiation treatment using three low γ-doses of 10, 20, and 30 kGy. The effect of irradiation was evaluated by Scanning Electron Microscopy (SEM), Fourier Transform Infrared Spectroscopy (FTIR), Differential Scanning Calorimetry (DSC), thermogravimetry (TG), chemiluminescence method (CL), migration studies, and antibacterial activity tests. It was found that in comparison with neat PLA, the gamma irradiation in the oxidative conditions of the PLA-based blends and biocomposites, causes modifications in the structure, morphology, and thermal properties of the materials depending on irradiation dose and the presence of natural additives such as rosemary and chitosan. It was established that under a gamma-irradiation treatment with dose of 10–20 kGy, the PLA materials showed minor changes in structure and properties being suitable for application in packaging and in addition after irradiation with such doses their antimicrobial activity against *Escherichia coli*, *Staphylococcus aureus*, and *Salmonella typhimurium* is improved.

## 1. Introduction

Irradiation of food, pharmaceuticals, and their packaging by ionizing radiation is a treatment frequently applied to improve safety and preservation of some materials. The ionizing radiation destroys dangerous contaminates in foods such as bacteria, viruses, fungi, and insects. It is applied for microbial reduction, to extend the shelf-life of irradiated food by effectively destroying micro-organisms responsible for spoilage and foodborne illness by sterilization of foods, and controlling insects and invasive pests, being applied for disinfestation of food and spices and also used for phytosanitary purposes, eliminating the risk of introducing foreign insects to other countries. Irradiation technology can be also applied to reduce allergenicity of allergic foods [1]. The process is not new, being used around for the past 60 years. Macronutrients (carbohydrates and lipids) are relatively stable against irradiation doses up to 10 kGy, but gamma irradiation affects the proteins leading to conformational changes, oxidation of amino acids, covalent bonds scission, and formation of protein-free radicals [2].

The irradiation is usually chosen for dairy products, processed food, beverages, pharmaceuticals, and medical device industries [3]. Irradiation of sensitive health care materials (hydrogels, herbs, drugs, etc.) and food items in a package is a special case, because any decomposition products by radiolysis that might be released during irradiation would migrate into food products [4]. Irradiation is approved as safe procedure by the U.S. Food and Drug Administration (FDA), the World Health Organization (WHO), the Centers for Disease Control and Prevention (CDC), and the U.S. Department of Agriculture (USDA) [5]. For example, such treatment uses doses up to 40 kGy with specific limits for each material [6]. The maximum accepted dose used on medical devices is 25 kGy, whereas a dose less than 10 kGy is usually applied to food. Packaging materials may be irradiated either prior to or after filling. This means that the levels of radiolysis products should be lower in food-packaging polymers as compared to medical devices [7]. In many countries of the European Union, only dried herbs, spices, and seasonings can be processed with irradiation and only at a specific dose, while in Brazil all foods are allowed to be treated with irradiation at any dose. In most countries, packaging materials are irradiated without the need for regulatory approval.

Biocomposites are very sensitive to gamma irradiation, which is used both for modification of their properties and for sterilization of polymeric materials [8,9,10]. Upon irradiation, the biopolymers experience various effects, such as crosslinking or reticulation, degradation, or scission of polymeric chains, depending on the structure of the polymer, irradiation dose, additive concentration, and irradiation conditions (atmosphere and temperature) [11]. A large number of major technological advances including irradiation have contributed to the development of higher strength, high-performance polymeric materials that provide excellent benefits for food packaging [12].

At slightly higher irradiation doses, the degradation could be the most important process which can be either avoided or desirable. Nevertheless, in most cases, when the service-life of biocomposite-based products is over, a huge quantity of waste is generated which has negative effects on the environment if the waste management measures are not applied. Under gamma irradiation, the materials disintegrate with a suitable rate allowing their integration in the environment. Such treatment can be applied to reduce the food packaging waste and/or food waste. Thus, gamma-irradiation technology could be an additional treatment of polymeric materials to promote their integration into the environment or even an alternative to the conventional procedures (mechanical and chemical) with advantages both economic and ecological points of view [13].

Biodegradable polymers and their blends and composites have been growing on the world market in recent decades. They possess well-known advantages that make them suitable for short-term applications, especially for food packaging being considered a solution for solving the growing problem of plastic waste management [14]. Unfortunately, composting and recycling of biodegradable products is an easy task only in the laboratory. Practically, the underdeveloped selective collection and sorting systems of compostable polymer waste cause many difficulties. For example, polylactic acid (PLA) is often indiscernible from polyethylene terephthalate (PET), and PET contamination with small amounts of PLA affects the recycled PET properties. The microbial degradation of PLA is limited to a few species of microorganisms; therefore, it results in excess of waste, which will be difficult to treat by biodegradation and it is necessary to find efficient recycling methods for such biodegradable polymers.

The irradiation of polymeric materials with ionizing radiation (γ-rays, X-rays, accelerated electrons, ion beams, etc.) leads to the formation of very reactive intermediates, free radicals, ions, and excited states which can experience disproportion, hydrogen abstraction, rearrangements, degradation, and/or the formation of new bonds depending on the structure of the polymer and the conditions of treatment. The cross-linking and degradation coexist during irradiation of many polymeric materials at certain irradiation doses.

PLA behavior under irradiation is still debatable, especially at low irradiation doses. Some literature reports found that no changes in structure are detectable, despite the observed effects of the ionizing irradiation on mechanical properties of PLA, or changes have been found only if the applied dose irradiation was up to 60 kGy or higher [15,16].

PLA crosslinking occurs by applying different types of irradiation, i.e., high energy electron beam or gamma irradiation and UV light which enables curing at mild conditions [15,16]. West et al. [17] established that, for doses up to 50 kGy, the performance of 3D-printed PLA is not largely affected, this being beneficial for applications in space and in medicine. At higher doses, decomposition processes became prevalent over cross-linking, so PLA degradation in waste streams will occur, avoiding environment pollution.

Naturally occurring polymers or their derivatives under normal conditions of irradiation undergo chain scission reaction [18]. In the case of radiation processing of polysaccharides, the degradation process can be performed at room temperature. Chitosan (CS) can be easily degraded by radiation in liquid state and in solid state [19]. Degradation occurs due to the breakage of the glycosidic bonds of polymers. Yoksan et al. (2004) [20] focused on the optimal conditions for γ-irradiation to reduce the molecular weight of CS, but when it still retains its chemical structure. The viscosity average molecular weight of the polymer decreased with increasing irradiation dose. The synthesis of chitosan oligomers via irradiation, both in solid and solution state, and changes with radiation dose were reported as being caused by main chain scissions [21].

The rosemary extract exhibits antioxidant, antibacterial, antifungal, antiviral, antimicrobial, anti-inflammatory, astringent, and spasmolytic activity and even anticancer properties, because of multiple contributions of its different bioactive compounds and its phytochemical composition rich in (poly)phenolic compounds, mainly diterpenoids such as carnosic acid and carnosol, flavonoids, etc., in plants. The major compounds imparting antioxidant activity of rosemary are: phenolic acids, phenolic diterpenes, and flavonoids such as 1, 8-cineol, α-pinene, β-caryophyllene, β-pinene, β-sitosterol, caffeic acid, camphene, carvacrol, carvone, epirosmanol, γ-terpinene, isorosmanol, limonene, linalool, myrcene, p-cymene rosmadial, rosmarinic acid, terpinen-4-ol, ursolic acid and verbenone either rosmarinic acid, an ester of caffeic acid, and 3,4-dihydroxyphenyllactic acid. Phenolic diterpenes, carnosol and carnosic acid were found to be the principal antioxidative components of the rosemary extract [22,23,24,25] and refs. therein. The rosemary extract also has a protective effect during irradiation (see below). Incorporation of natural additives in PLA-based materials is performed to confer new characteristics to materials as resulted from our previous studies [10,24,25,26,27]. It was found that by adjusting the ratio of components in recipes, it is possible for either flexible or rigid products to be obtained. Chitosan and rosehip seed oil or rosemary extract as bioactive antimicrobial/antioxidant agents acted synergistically, increasing the shelf life of the packaged products. These biocomposites showed improved processability and physical–mechanical, thermal, water vapor barrier, antioxidant, and antimicrobial activities, being suitable for use in food packaging. The presence of natural compounds in the PLA-based biocomposites determines an acceleration of fungal degradation [28,29].

The purpose of this paper is to establish the effect of the gamma irradiation of low doses on the two types of newly elaborated polymeric materials based on polylactic acid (PLA)/rosemary ethanolic extract (R) blends and on polyethylene glycol (PEG)-plasticized polylactic acid (PLA), chitosan (CS), and R biocomposites, to assess their suitability for packaging application, when such gamma-irradiation treatment is applied. Such materials, characterized in detail in our previous papers, were subjected to gamma-irradiation treatment using three γ-doses of 10, 20, and 30 kGy. It is expected that the treatment by gamma irradiation using low doses (~10 kGy) will improve the food safety and preservation of the products packed in such materials, and to conclude whether, at higher doses (≥30 KGy), the deterioration of their properties is more advanced so it may be considered that their corresponding packaging waste will be disintegrated. To evaluate the gamma-irradiation effect on the new PLA-based materials, several sensitive methods were selected to evidence structural and morphological changes, variation in the spontaneous emission of light from an electronically excited state of a species produced by chemical reactions occurring during gamma irradiation, variation in thermal properties, bioactive compounds release, and antibacterial properties such as scanning electron microscopy (SEM), Fourier Transform Infrared Spectroscopy (FTIR), differential scanning calorimetry (DSC), thermogravimetry (TG), chemiluminescence method (CL), bioactive compounds release study, and migration kinetics and antibacterial activity tests.

## 2. Materials and Methods

### 2.1. Materials

Polylactic acid (PLA) obtained from renewable resources, transparent (trade name: PLA 2002D), from NatureWorks LLC, Minneapolis, MN, USA has a melt flow index of 5–7 g/10 min (conditions 210 °C/2.16 kg) and a content of 96% l-lactide and 4% D isomer. Average molecular weight determined by GPC was 4475 kDa. According to firma specifications and the literature data, it has a density of 1.25 g/cm^3^, melting point of 152 °C, and glass transition temperature of 58 °C. The crystallinity depends on isomers content and thermal history, water permeability at 25 °C is 172 g/m^2^ per day, and percentage of biodegradation/mineralization in laboratory conditions was 100% [30].

Chitosan medium molecular weight (CS) with 200–800 cP viscosity in 1% acetic acid, M_W_ = 190–300 kDa and 75–85% deacetylation degree was provided and used as received from Sigma-Aldrich, Steinheim am Albuch, Germany.

Rosemary extract (R) in powder form was obtained following a previously reported procedure by the solvent extraction method in a Soxhlet unit [31]. Rosemary leaves were collected from local farms, dried at ambient temperature, and subsequently milled (Laboratory of Radiation Chemistry, INCDIE—ICPE CA. Bucharest, Romania). Ethanol was used as an extraction solvent. After obtaining the rosemary extract in an ethanol solution, the powder was separated by precipitation with water. A greenish-yellow fine powder was obtained and stored in a desiccator to avoid the absorption of moisture. Its main components were: carnosol, carnosic acid, and rosmarinic acid [32]. The total phenols content was 112.5 mg GAE (Gallic acid equivalent)/g dw (dry weight), while the total flavonoid content was 261.5 (mg QE (Quercetin Equivalents)/g dw) [26].

Poly(ethylene glycol) (PEG) BioUltra 4.000 from Sigma-Aldrich Steinheim am Albuch, Heidenheim, Germany was used as the plasticizer.

### 2.2. Biocomposite Preparation

PLA-based blends/biocomposites were prepared using different amounts of rosemary extract or/and chitosan by incorporating them into the PLA matrix in melt state using a fully automated laboratory Brabender station (Brabender^®^ Plasti-Corder^®^ Lab-Station/Lab-Station EC, Brabender GmbH & Co. KG, Duisburg, Germany). The processing conditions were temperature of 165 °C for 10 min, at a rotor speed of 60 rpm. Here, both PLA/R blends were studied containing 0.25–0.75 wt% R and PLA/PEG/CS/R biocomposite systems containing 20 wt% PEG, 3 wt%, and 6 wt% CS and 0.5 wt% (R)—Table 1. More details on the studied materials’ preparation and their characteristics/properties are found in the previous paper [25].

### 2.3. Radiochemical Processing

The γ-radiation processing was performed by a γ-irradiator M-38 GAMMATOR (Washington D.C., WA, USA), using a ^137^Cs source in air at room temperature at a dose rate of 0.4 kGy/h, samples being subjected to γ-doses of 10, 20, and 30 kGy.

### 2.4. Investigation Methods

#### 2.4.1. Scanning Electron Microscopy (SEM)

The SEM studies were performed on samples fixed on copper supports. The surface was examined by using an Environmental Scanning Electron Microscope (ESEM) type Quanta 200 instrument (FEI Company, Hillsboro, TX, USA) operating at 25 kV with a Low Vacuum Secondary Electron (LFD) detector.

#### 2.4.2. ATR–FTIR Spectroscopy

The ATR–FTIR spectra were recorded on a Bruker VERTEX 70 spectrometer (Ettlingen, Germany) with a 4 cm^−1^ resolution. The background and sample spectra were obtained in the 600–4000 cm^−1^ wavenumber range. Spectral processing was achieved with the OPUS and ORIGIN programs.

#### 2.4.3. Differential Scanning Calorimetry (DSC)

DSC of irradiated/non-irradiated PLA/R blends and PLA/PEG/CS/R biocomposites was performed under air atmosphere by means of a DSC 823^e^ Dynamic Scanning Calorimeter (Mettler Toledo, Greifensee, Switzerland). Samples of ~2–5 mg were heated up from room temperature (which was below the glass transition) to 200 °C (above the melting temperature of the materials). After the first heating run, each sample was kept for 2 min at 200 °C and then cooled down to room temperature with a cooling rate of 10 °C/min and reheated up to 200 °C with a heating rate of 10 °C/min. An empty aluminum crucible was used as a reference.

The thermal properties determined from the DSC curves were the glass transition temperature (T_g_), cold crystallization temperature (T_cc_), and melting temperature (T_m_) with an overall accuracy of ±0.5 °C.

#### 2.4.4. Thermogravimetry (TG)

TG experiments were carried out with a DuPont Instrument device (Wilmington, DE, USA) Thermal Analyst 2000/2100 coupled to a module 951 Thermogravimetric Analyzer, using a heating rate of 10 °C/min under nitrogen atmosphere with a purge rate of 50 mL/min, between room temperature (25 °C) and 500 °C.

#### 2.4.5. Chemiluminescence (CL)

The LUMIPOL 3 unit (SAS, Bratislava, Slovakia) chemiluminescence spectrometer was used for the recording of non-isothermal emission intensity dependence on temperature on film samples with small weights not exceeding 5 mg. The selected temperature range starts from room temperature, ending at 250 °C. The measured temperatures had a low error (±0.5 °C). Heating rates were 2, 3.5, 5, and 10 °C/min. CL determination was carried out in air under static conditions. The CL intensity values were normalized to sample mass for their reliable comparison. The activation energy of the oxidation process was determined using the Kissinger method [33,34].

#### 2.4.6. Bioactive Compounds Release and Migration Kinetics Investigation

The polyethylene glycol (PEG)-plasticized PLA/chitosan/powdered rosemary extract biocomposites of various compositions (3 wt% CS or 6 wt% CS) irradiated at dose of 30 kGy were studied in terms of migration kinetics in conditions that mimic storage at ambient temperature for unlimited duration, as for a minimum of 10 days (14 days) at 40 °C, using a 50% aqueous ethanol (*v/v*) solution as modified D1 food simulant [35]. This is considered the most severe aqueous food simulant for dairy products and alcohol containing foods [36]. Detailed descriptions of the experimental procedure and kinetic interpretation of the data can be found in [25,26]. Concentrations of the released active components were calculated based on the calibration curves performed for the main active components of rosemary extract as previously determined [25,26] and according to the literature data [37,38,39].

#### 2.4.7. Antibacterial Activity

The antibacterial efficiency testing was performed against two Gram-negative strains, *Escherichia coli* (ATCC 25922) and *Staphylococcus aureus* (Staphylococcus aureus ATCC 25923), and one Gram-positive strain *Salmonella typhimurium* (ATCC 14028) and this consisted of the following steps:

1. Sterilization of the samples, performed in an autoclave at 110 °C, 0.5 bars for 20 min.
2. Preparation of ATCC cultures by: seeding an average pre-enrichment and incubation at 37 °C for 24 h; counting the colonies in 0.1 mL culture by selective culture medium separation. Seeding of 0.1 mL bacterial culture ATCC using sterile swab sample surfaces. Both samples and reference (blank as plastic food foil) were inoculated with 10 µL inoculum ATCC containing 10^5^ UFC/mL.
3. Incubation of samples contaminated with the ATCC for 24 h at 25 °C, in the dark, in sterilized glass containers, was repeated after 24 h of incubation; using a sterile swab, the inoculated surface was cleaned with physiological saline. Then, the surfaces were seeded with the selective media such as XLD for *Salmonella*, Rambach for *E. coli*, and Chapmann for *Staphlococcus aureus*.
4. Identifying target germs was undertaken following standardized methods of bacteriology procedures, according to standards in force: SR ISO 16649 for *E. coli*; SR EN ISO 6579 for *Salmonella* sp.; and SR EN ISO 6888 for *Staphylococcus aureus*.

The colonies were counted after 24 h of contact of after incubation at 37 °C.

## 3. Results and Discussion

### 3.1. Scanning Electron Microscopy (SEM)

**Figure 1:**
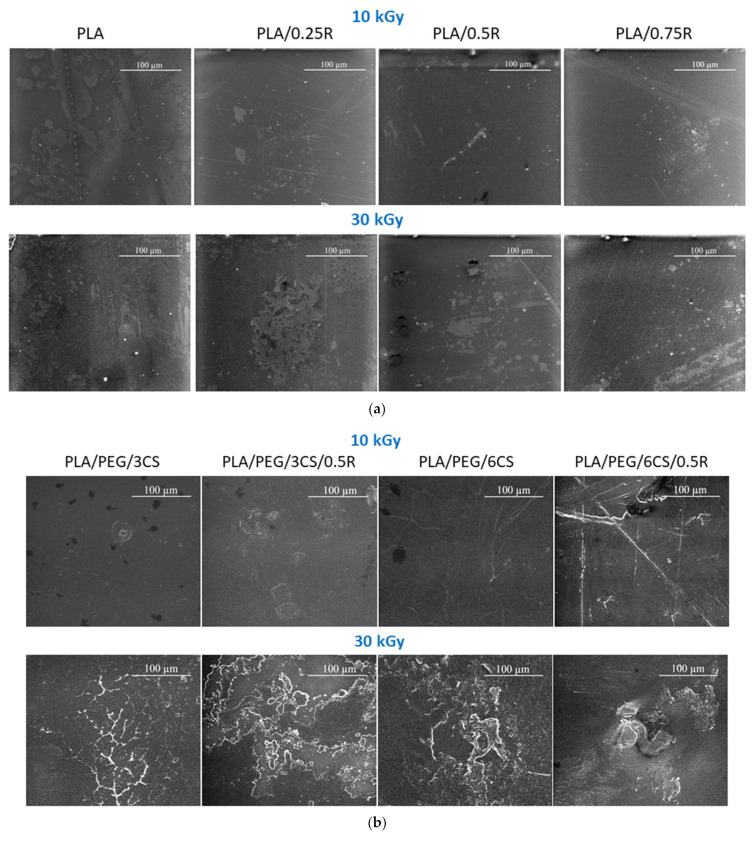
SEM pictures of the surface of the PLA and PLA/R blends (**a**), and of PLA/PEG/CS and PLA/PEG/CS/R biocomposites (**b**) at various gamma irradiation doses; magnification 1000×, scale—100 µm.

The effect of γ-irradiation on the surface morphologies of the PLA-based samples was examined using SEM. In the SEM images of Figure 1a, the surface morphologies (at a scale of 100 µm) of PLA and PLA/R blends before and after irradiation with doses of 10 and 30 kGy, respectively, were shown. As it is observed, the surface of the samples’ remains almost smooth without major damage after irradiation with a dose of 10 kGy, in accordance with literature results [40,41]. This aspect remains unchanged also for a 20 kGy irradiation dose. At a dose of 30 kGy, the surfaces of all samples are heterogeneous, but cracks are not present. Similar observations were found in the case of plasticized samples. The addition of CS in the composition of biocomposites’ induced some modifications at their surface with the increase in γ-dose, mainly at 30 kGy (Figure 1b). The heterogeneity of the surfaces’ also increased with increasing CS content and irradiated dose—Figure 1b, showing scratches and minor cracks.

### 3.2. FTIR Spectroscopy

Some authors have reported that gamma irradiation did not induce any detectable change in intensity or any wavenumber shift of FTIR spectra bands of neat PLA, despite the observed effects of the ionizing irradiation on mechanical properties of PLA [42,43], or changes have been found only if the applied dose irradiation was up to >60 kGy [41].

However, for complex materials and depending on radiation doses, changes have been found [44]. The samples under study showed shifts in intensity and band positions of the main components of the biocomposites, namely PLA and PEG because of interaction between components [25]. The C=O band of PLA shifts to a lower wavenumber in the PEG-plasticized PLA spectrum due to the possible strong hydrogen bonding with hydroxyl end-groups of PEG. The characteristic wide absorption band at 2730–2984 cm^−1^, assigned to CH_2_-stretching vibration in carbonyl compounds, shows multiple overlapped bands with narrow band intensity upon incorporation of CS and the band intensity at 2946 cm^−1^ decreased with the addition of CS. It is expected that the biocomposites have a different behavior to gamma irradiation.

**Figure 2:**
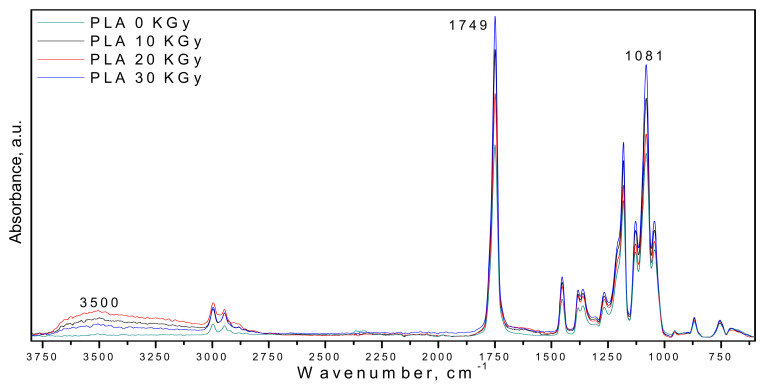
FTIR spectra of PLA at gamma irradiation doses of 0, 10, 20, and 30 kGy.

Hence, no changes were observed in the chemical structure of the γ-ray-treated PLA sample, irrespective of applied γ-irradiation doses (10–30 kGy)—Figure 2. PLA shows characteristic stretching frequencies for C=O, –CH_3_ asymmetric, –CH_3_ symmetric, and C–O, at 1746, 2995, 2946, and 1080 cm^−1^, respectively. Bending frequencies for –CH_3_ asymmetric and –CH_3_ symmetric were identified at 1452 and 1361 cm^−1^, respectively. In the region of 1600–1900 cm^−1^, the band located at 1758 (1749) cm^−1^ assigned to ester groups decreases as a function of radiation dose; therefore, the irradiation of PLA occurs due to oxidation reactions of ester groups leading to formation of hydroperoxides or alcohols, the hydroxyl groups being present in the region 3300–3600 cm^−1^ as a large absorption band centered at 3500 cm^−1^ and its intensity increases with radiation dose—see Figure 2.

**Figure 3:**
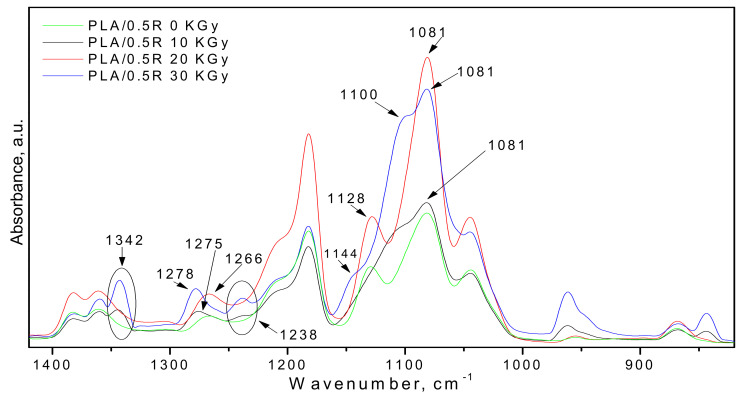
FT-IR spectra of PLA/0.5R blend at three different gamma irradiation doses (10, 20, and 30 kGy) in comparison with the non-irradiated one.

Comparing the spectra of the non-irradiated samples of PLA/R films (PLA/0.25R, PLA/0.5R, and PLA/0.75R) with those γ-irradiated, some significant modifications occur after gamma irradiation, especially in the 1400–800 cm^−1^ region—Figure 3. Thus, after sample irradiation, two new absorption bands appear at 1342 and 1238 cm^−1^ which are assigned to the CH and CH_2_ bending twisting vibrations, respectively. Moreover, in the spectra of non-irradiated samples, the band from 1128 cm^−1^ (attributed to the CH_3_ rocking or C–O stretching) was shifted to 1100 cm^−1^ and changed in a shoulder which is stronger, as evidenced in the case of the samples irradiated at a dose of 30 kGy (blue line).

PEG plasticized PLA shows almost the same absorption bands as pristine PLA (not given). The FTIR spectrum of the PEG-plasticized PLA clearly shows the characteristic absorption bands in the regions of 3350–3450 cm^−1^, 2750–3000 cm^−1^, and at 1645 cm^−1^ due to O–H bending and stretching vibration, C–H asymmetric stretching vibration, and C=O stretching of ester bonds, respectively. A broad band was observed at 3446 cm^−1^ for PEG, which corresponds to the terminal hydroxyl group [45]. There are many studies focused on variation in the structures and properties of the biomaterials containing PEG that underwent gamma irradiation [46]. Gamma irradiation of PEG with high doses [46,47,48] led to variation in intensity of 1720 and 1680 cm^−1^ absorption bands assigned to C–O or C=O groups with a gradual increase in intensity of these absorption bands with an increase in irradiation dose, indicating the formation of a carbonyl group upon irradiation. In FTIR spectrum, bands are also present at 3450 cm^−1^ for the strong OH group, 2960 cm^−1^ medium for the CH_2_/CH_3_ group, 1450 cm^−1^ medium for the CH_3_ group, 1380 cm^−1^ medium assigned to the CH_3_/CH_2_ group, and 1180 cm^−1^ medium for CH_2_/CH group. By ESR it was found that by irradiation of PEG, the free radicals of the type ~CHO. ~(I) and ~CH_2_O (II) were formed. The small changes in physico-chemical and pharmacological properties of PEGs were found to be similar to those of control and heat-sterilized samples [49].

Because of small changes evidenced in many irradiated biomaterials, the 25 kGy is considered the most common dose of gamma irradiation for serialization of biomedical products containing PEG [47,48].

**Figure 4:**
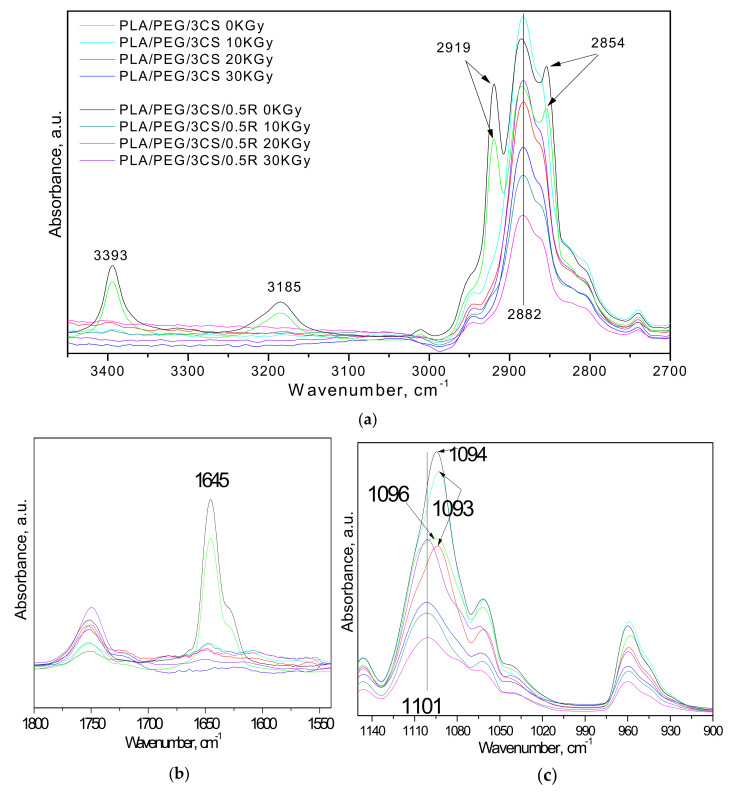
FT-IR spectra of the PLA/PEG/3 CS with/without rosemary extract powder (R), at 10, 20, and 30 kGy irradiation doses, respectively, in comparison with the non-irradiated ones in different spectral regions of: (**a**) 2700–3500 cm^−1^, (**b**) 1500–1800 cm^−1^, and (**c**) 900–1150 cm^−1^.

**Figure 5:**
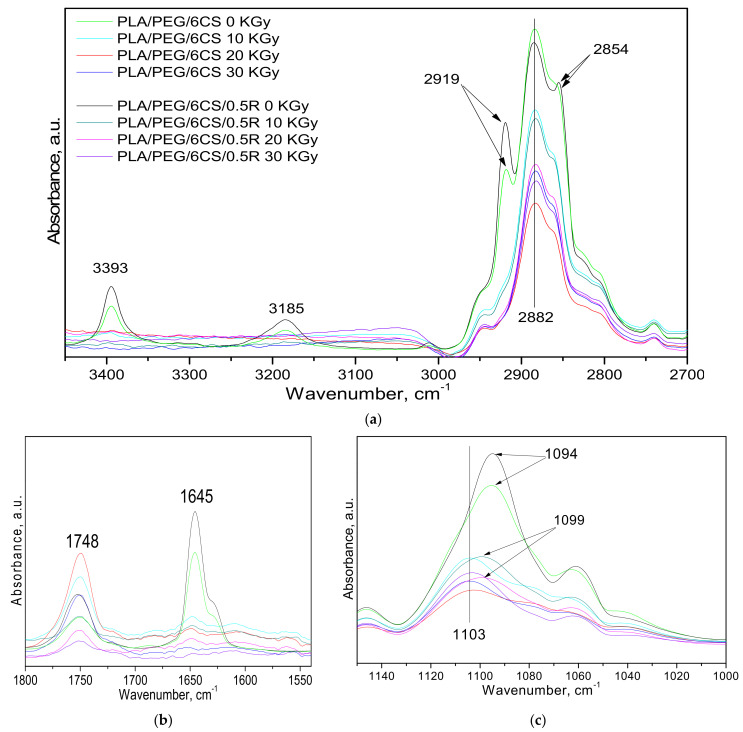
FT-IR spectra of the PLA/PEG/6 CS with and without rosemary extract powder (R) at three different irradiation doses (10, 20, and 30 kGy) in comparison with the non-irradiated ones for three spectral regions: (**a**) 2700–3500 cm^−1^, (**b**) 1500–1800 cm^−1^, and (**c**) 1000–1150 cm^−1^.

The gamma irradiation of PLA/PEG/CS/0.5R biocomposites induced some modifications in their infrared spectra, as observed from Figure 4 and Figure 5 corresponding to the biocomposites with different CS contents of 3 wt% CS and 6 wt% CS, respectively.

Thus, after irradiation, their FTIR spectra did not show more absorption bands at 3393, 3185, 2919, 2854 (Figure 4a and Figure 5a), and 1645 cm^−1^, which are attributed to the chitosan (Figure 4b and Figure 5b) [50].

Rezanejad et al. [51] published a detailed FTIR study on gamma irradiation of rosemary, indicating the main absorption bands in its FTIR spectra, found also in our spectra (Figure 2, Figure 3, Figure 4 and Figure 5), namely: broad absorption band at 3415 cm^−1^, corresponding to OH stretching bands of alcohols and/or carboxylic acid vibrations, the 2929 and 2854 cm^−1^, assigned to vibration of the –CH_3_ asymmetric stretching and symmetric stretching absorption band of the methylene group vibration, respectively. The 1636, 1453, 1376, 1262, 1039, and 603 cm^−1^ bands are assigned to the bond vibrations of the asymmetrical carboxylic acid and C=O stretching vibration, C–N stretching, symmetrical carboxylic acid group, C–O stretching vibrations (amide) and phenyl groups and of the C–O stretching, and at last attributed to C–O stretching vibrations of mono-, oligo-, and carbohydrates, respectively. The bands in the 1617–1672 cm^−1^ wavenumber region also show a 1659 cm^−1^ shoulder assigned to C=O stretching vibration C–N stretching, and COO^−^ asymmetric stretching. After irradiation, crude rosemary had similar patterns of FTIR spectra, typical of phenol compounds, without any notable changes in the key bands or functional groups status. This is in accordance with chemiluminescence results.

In the case of the PLA/PEG/3CS sample (Figure 4c), the band at 1094 cm^−1^ (associated with the stretching vibration of C–O–C from PLA) was shifted to 1101 cm^−1^ only after the irradiation with a 30 kGy dose. When the rosemary powder (0.5R) was added to the biocomposites, the shift in this absorption band was observed also at lower doses (10 and 20 kGy). When a high quantity of chitosan was presented in the PLA/PEG/6CS biocomposite (Figure 5), the shift after irradiation of the 1094 cm^−1^ band to a higher wavenumber (1103 cm^−1^) occurred irrespective of the presence or absence of rosemary powder extract (0.5R). This suggested various interactions between components of biocomposites after irradiation due to modifications in the structure of some of them.

Some changes in intensity after irradiation were presented for hydroxyl and carbonyl absorption bands. In the 3400–3600 cm^−1^ spectral region, the FTIR spectra of the samples present a large absorption band centered at 3506 cm^−1^ in the case of PLA, which corresponds to OH groups in alcohols, hydroperoxides, or carboxylic acids. After exposure to gamma radiation, this band becomes stronger in intensity and increased with radiation dose. According to results previously reported by other authors, for the PLA gamma irradiation [44,52], this change can be explained by the formation of hydroperoxide derivatives which degrade in compounds containing carboxylic acid and di-ketone end groups.

PLA crosslinking also occurs by applying different types of irradiation [16], this leading to changes in mechanical and thermal properties. Sensitivity to irradiation is mainly due to the presence of methine hydrogen atoms (3185 cm^−1^) along the polymer chain as well as the possibility of introducing functional end-groups. However, not only crosslinking but also other reactions such as formation of active species as radicals and also branching and chain scission proceed upon irradiation. However, in the case of PLA/0.25R, PLA/0.5R, PLA/0.75R, and PLA/PEG/6CS/0.5R samples, the 1748 cm^−1^ characteristic band absorption decreases as a function of radiation dose, as the R extract has a protective action against irradiation. This result is in accordance with those obtained by chemilumiscence method (see below). The literature data demonstrated that gamma irradiation leads to oxidation reactions inducing the formation of hydroxyl groups which can be present in hydroperoxides or alcohols [45]. As it was already mentioned, in the radiation processing of polysaccharides including CS, the degradation process occurs at room temperature [19] by the breakage of the glycosidic bonds (bands at 1130–1160 and 1155 cm^−1^) of polymers reducing molecular weight and viscosity. These characteristic bands showed reduced intensities or disappeared from the FTIR spectra of biocomposites. The characteristic bands of chitosan were observed at 3000~3900 cm^−1^ (OH, NH_2_), 2867 cm^−1^ (–CH stretching), 1655 cm^−1^ (–CONH–, amide I), 1554 cm^−1^ (–NH, amide II), 1573 cm^−1^ (C–N stretching), 1322 cm^−1^ (amide III bond), and 1107 cm^−1^ (C–O stretching vibration). Wenwei et al. [53] found some chemical changes in chitosan induced by γ-ray irradiation. It was found that the NH– group is more sensitive to irradiation than the –NHCOCH_3_ group and, moreover, the hydroxyl group increases with increasing radiation dose while the –C–O–C– group decreases, but no evidence for carbonyl formation was observed. Reduction in viscosity and molecular weight of CS by irradiation is useful in medical applications. Size, crystallinity, and thermal properties of the chitosan microparticles did not change significantly after γ-irradiation. γ-irradiated microparticles exhibited a slightly higher drug release rate and low swelling capacity than the non-irradiated microparticles. By spectral characterization, it was shown that γ-irradiation of chitosan microparticles induced neither crosslinking nor formation of new groups in the chitosan matrix; the gamma rays induced only one kind of free radical in the chitosan matrix or reduction in average molecular weight [20,54,55].

### 3.3. Thermal Properties

#### 3.3.1. Differential Scanning Calorimetry (DSC) Results

Thermal behavior of the non-irradiated PEG-plasticized PLA/CS/R biocomposites has been detailed presented in our previous paper [27]. Differential scanning calorimetry, combined with chemiluminescence and coupled thermogravimetry-Fourier Transform Infrared spectroscopy–mass spectroscopy methods were applied to test both the thermal behavior and to establish the composition–properties relationship of these materials. It was found that the addition of CS in the PLA matrix shifted the T_g_ to slightly higher values, while the cold crystallization temperature of PLA-based biocomposites to lower temperatures showing that the CS can promote the crystallization of PLA. Melting temperature is not changed. By chemiluminescence study, it was established that the rosemary ethanolic extract is a good stabilizer for thermoxidative degradation of PLA. The highest onset (Tonset), maximum (T_max_), and decomposition temperatures corresponding to 10 wt% (T10) and 20 wt% (T20) mass loss were found for the PLA/PEG/6CS and PLA/PEG/6CS/R biocomposites. The PEG-plasticized PLA/CS/R materials show good thermal properties and most of thermal degradation products are non-toxics; therefore, they are recommended for both medical and food packaging applications. Here, the irradiation effect on some thermal characteristics of these PLA-based materials is presented.

**Figure 6:**
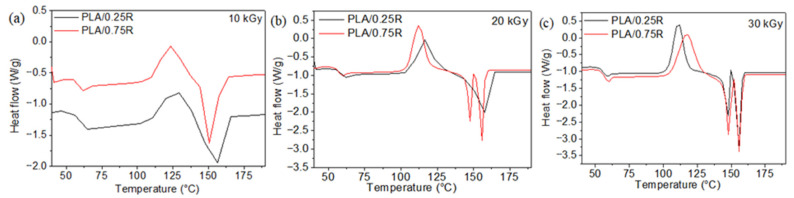
DSC curves of the PLA/0.25R and PLA/0.75R samples irradiated at 10 (**a**), 20 kGy (**b**), and 30 kGy (**c**). Recorded in the 2nd heating run.

**Figure 7:**
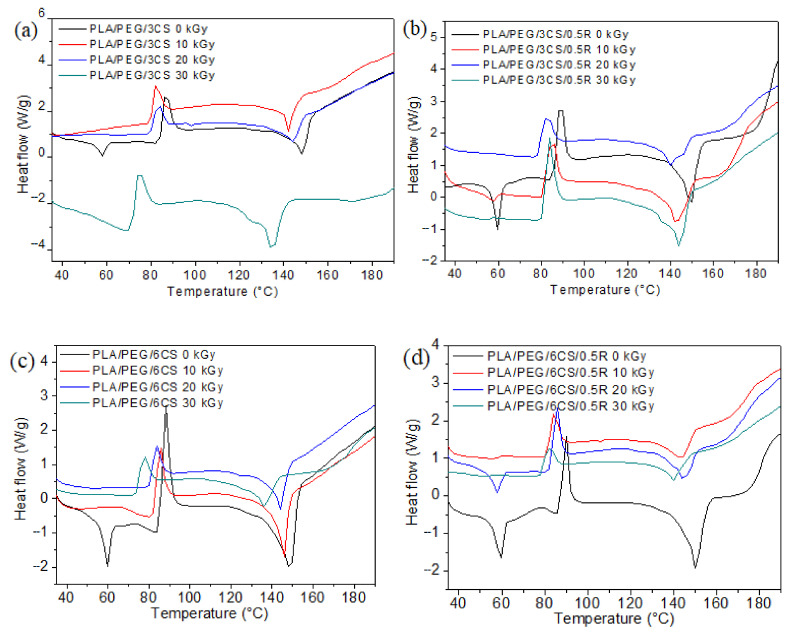
DSC curves for of PLA/PEG/3CS (**a**), PLA/PEG/3CS/0.5R (**b**), PLA/PEG/6CS (**c**), and PLA/PEG/6CS/0.5R (**d**) samples irradiated at 10, 20, and 30 kGy in comparison with non-irradiated samples. Recorded in the 2nd heating run.

The thermal properties evaluated from DSC curves in Figure 6 and Figure 7 are summarized in Table 2.

As observed from Table 2, the glass transition decreases for all studied samples after irradiation, especially with doses of 20 and 30 kGy. This decrease in T_g_ with irradiation dose can be explained by the radiation-induced chain scission and formation of short chain fragments. This is in accordance with other studies [16,56]. Radiation-induced scission of the CS chains resulted in a lower glass transition temperature (T_g_) at an irradiation dose of 25 kGy in air. Radiation-induced scission of the chitosan chains resulted in a lower glass transition temperature (T_g_), indicative of higher segmental mobility [57].

The crystallization temperatures (T_cc_) of all samples decrease with the increase in irradiation dose, especially at 30 kGy due to the easier crystallization of lower molecular weight chains formed as an effect of radiation-induced scission [56]. The rosemary extract effect on T_cc_ values consists of increasing them with content of R from 121.5 °C at PLA/0.25R to 126.5 °C at PLA/0.75R, respectively. After irradiation, Tcc values show a decrease with radiation dose for all studied biocomposites and high values with increased R concentration are still observed. The presence of rosemary extract in the PLA matrix inhibits the reaction of free radicals with oxygen during radiation processing, because of the improved resistance to degradation [58,59].

The melting temperatures regularly decrease upon irradiation for the majority of the samples. The decrease in melting temperatures with increasing irradiation dose is attributed to the formation of PLA fragments with lower molecular weight and to the decrease in crystalline perfections. Both changes result in greater mobility of macromolecules [17,44].

Moreover, before gamma irradiation, the DSC curves show the presence of a single melting peak at 149–156 °C. After irradiation, melting occurs as double melting peaks which may be explained by re-organization during irradiation and occurrence of different kinds of crystals. The irradiated neat PLA shows also two endothermic peaks of melting at 150.3 and 156.5 °C (due to the melting of different types of PLA crystals).

#### 3.3.2. Thermogravimetry (TG) Results

**Figure 8:**
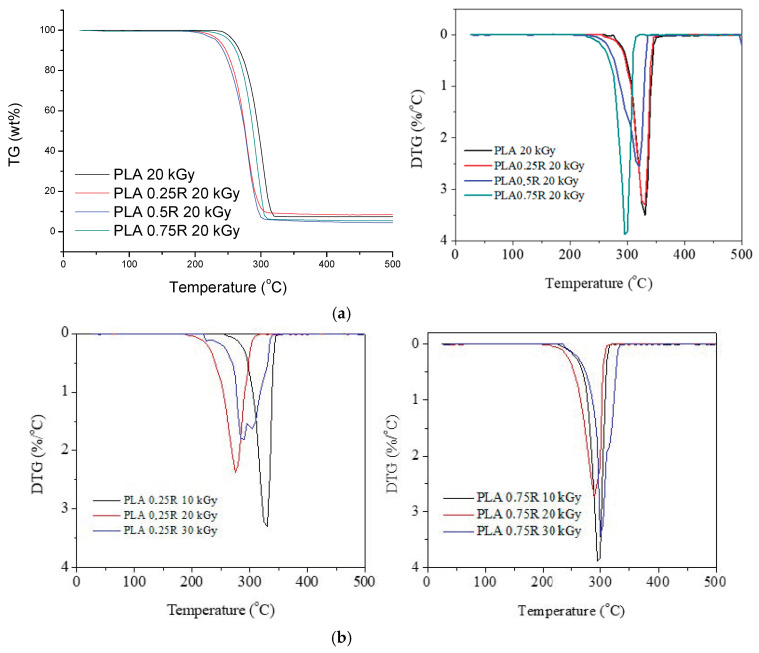
TG/DTG curves of the PLA/R blends with different R content irradiated with a gamma dose of 20 kGy (**a**); DTG curves of the PLA/0.25R and PLA/0.75R at different gamma irradiation doses (**b**).

**Figure 9:**
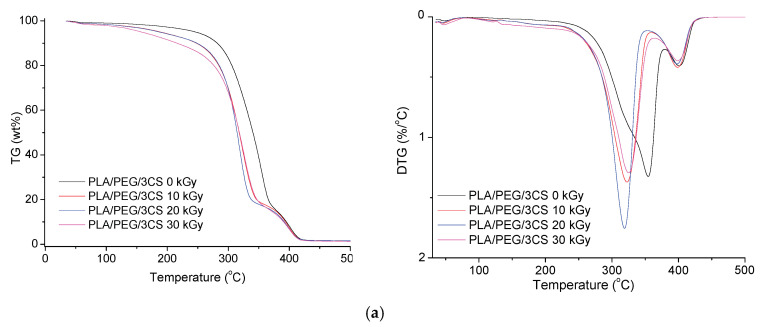

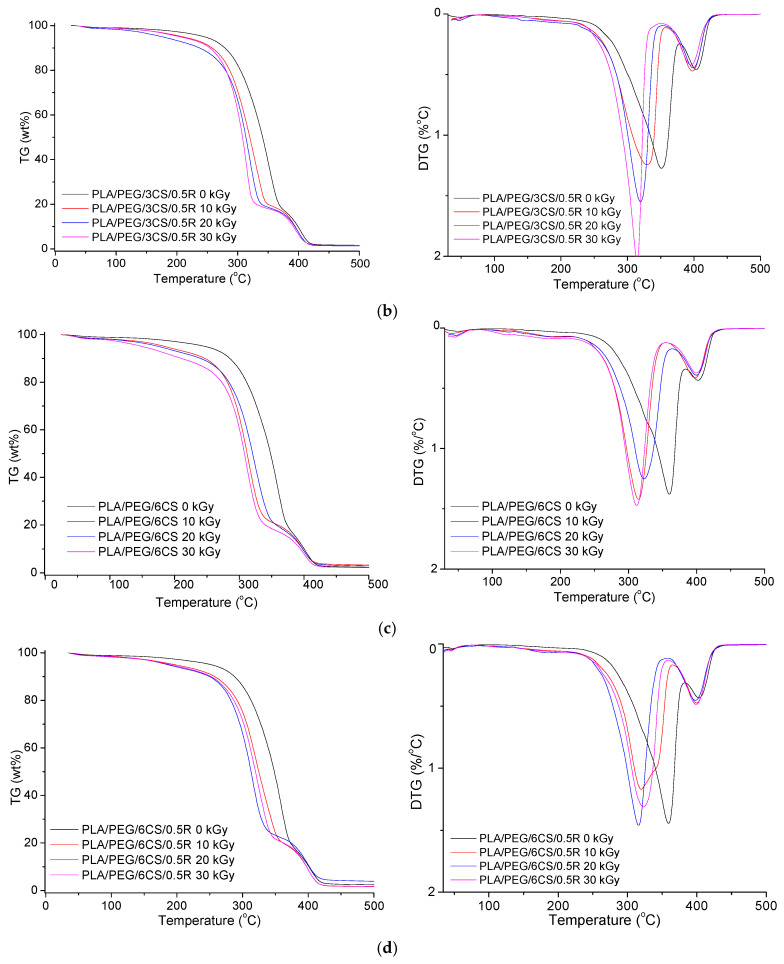
TG (**left**) and DTG (**right**) curves of the PLA/PEG/CS (**a**,**c**) and PLA/PEG/CS/R (**b**,**d**) non-irradiated and irradiated at 10, 20, and 30 kGy.

The TG/derivative of TG (DTG) curves of PLA/R blends and PLA/PEG/CS/R biocomposites irradiated at three doses (10, 20, 30 kGy) are shown in Figure 8 and Figure 9, respectively. It can be observed that the characteristic degradation temperatures (Table 3) decreased after gamma irradiation, the results being in accordance with the DSC data.

For R loaded into PLA samples, the DTG curves show a single decomposition peak but after irradiation two decomposition peaks or peaks with shoulders are found—Figure 8a,b, and was also found in DSC curves of irradiated samples. Their characteristic temperatures and mass losses have a slight tendency to decrease after irradiation with 10 and 20 kGy irradiation doses and show a slight increase again at 30 kGy; the PLA crosslinking likely also had a role during irradiation. It seems that the sample containing 0.75 wt%R has a better stability to irradiation (Figure 8b), as compared with the PLA/0.25R blend. The TG/DTG curves of the biocomposites containing CS and CS/0.5%R show two peaks of decomposition whose characteristic temperatures decrease with the increase in radiation dose; therefore their thermal stability is decreased by irradiation (Figure 9a–d). The decrease is smaller for biocomposites containing R.

Residual weight loss (Δw) values generally decrease with the increased radiation dose as was also found in other studies [17].

### 3.4. Chemiluminescence (CL)

The characterization of the radiation effects on studied PLA-based blends and biocomosites is based on the contribution of mixing components and by the reactions of specific intermediates [60]. Under gamma irradiation, the scission of macromolecular chains of the poly(L-lactic acid) takes place randomly, whose effect is the sharp decrease in the numerical average molecular weight [61,62]. On exposure to gamma radiation, several free radicals centered on carbon atoms are formed when volatile products are released or polymeric configurations are restored [63]. The depolymerization of PLA onto lactone [11,64] and the formation of carbonyl radicals from PEG [65] contribute to the further structures, similarly with the crosslinking PLA-PEG-PLA networks [66].

The addition of phenolic antioxidants such as rosemary extract powder usually increases the thermal and radiation stabilities by scavenging degradation precursors [67]. The contribution revealed by the components of this natural stabilizing mixture is described by means of the differences between the CL intensities recorded for stabilized systems.

**Figure 10:**
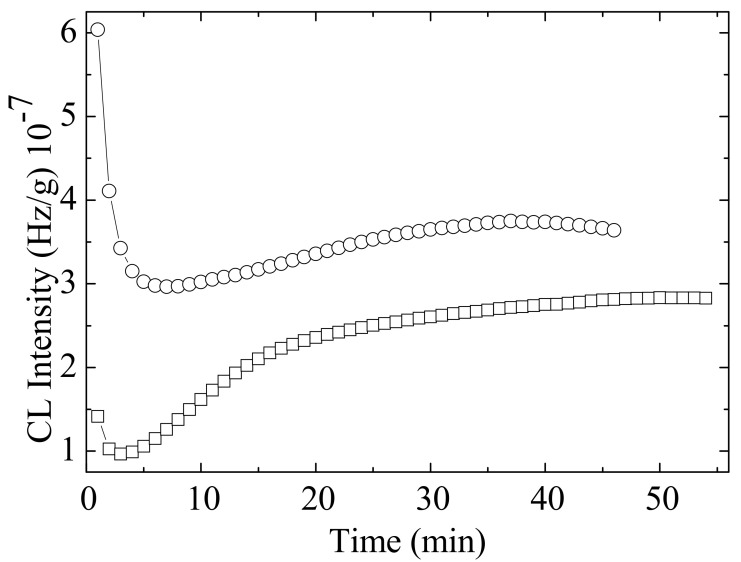
Isothermal CL spectra recorded on irradiated PLA/PEG/3CS samples without (○) and with 0.5 wt% rosemary extract (□); temperature: 180 °C; dose rate: 1.2 kGy/h.

The profiles for PEG-plasticized PLA mixed with R biocomposites are different as the concentrations of radicals are unlike. The scission of macromolecules during irradiation creates high local amount of carbon-centered radicals which can be caught by antioxidant molecules. Accordingly, the short time (about 10 min) of the disappearance of radicals illustrated by the firm decrease in the CL emission describes the efficient action of phenolic molecules (Figure 10). The trend of smooth increase in CL signal indicates the high protection activity of additives against oxidation.

The variation of CL intensity over time (Figure 10) is proof for the accelerated degradation of PLA/PEG/CS samples that is delayed by the active components of rosemary extract. However, the period over which oxidation is hindered is very short, about 4 min at 180 °C. It may be assumed that this effect would be more relevant at lower temperatures, where the material degradation occurs slowly.

**Figure 11:**
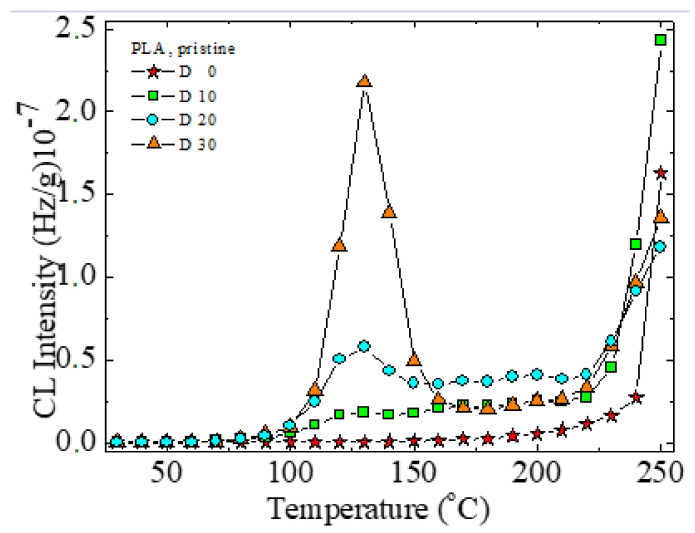
Non-isothermal CL spectra for degrading PLA subjected to various gamma irradiation doses.

In PLA, the formation of peroxides starts relatively early. The temperature of 126 °C indicates a fast decomposition of hydroperoxides (Figure 11). The accumulation of free radicals during radiolysis leads to a higher peak at this temperature, followed by new scission providing radicals for oxidation. The main degradation step is visible after 200 °C, when the majority of weak bonds are broken.

**Figure 12:**
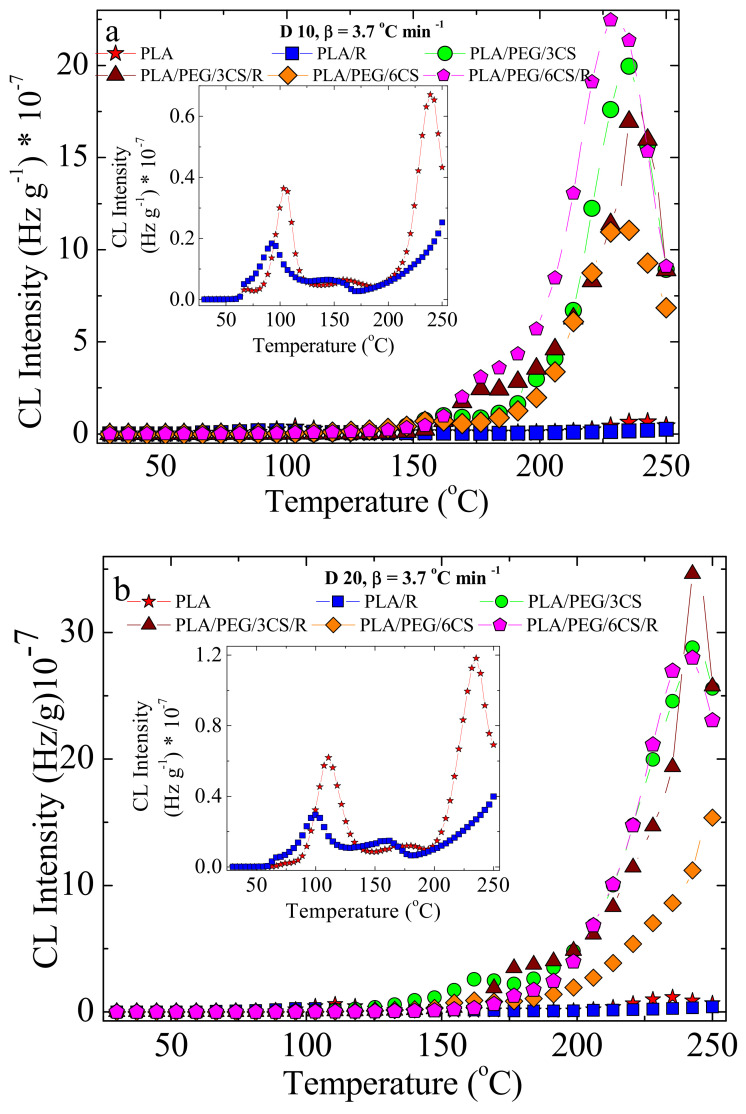

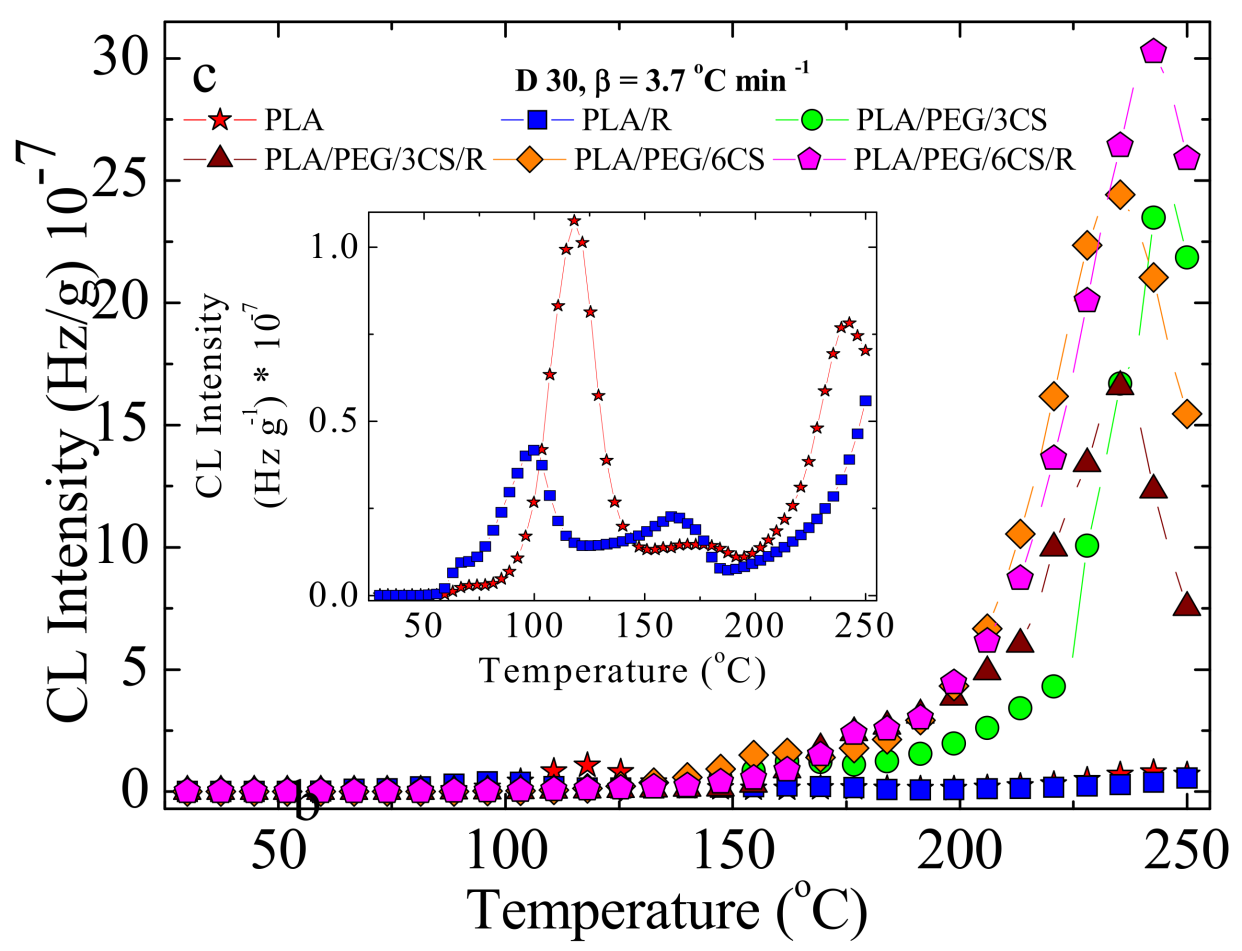
Nonisothermal CL spectra recorded on PLA samples stabilized with rosemary extract. Heating rate: 3.7 °C/min; dose rate: 1.2 kGy/h. Additive concentrations: (**a**) 0.25 wt%; (**b**) 0.50 wt%; (**c**) 0.75 wt%. (red stars) 0 kGy; (□) 10 kGy; (○) 20 kGy; (Δ) 30 kGy.

The modification in the rosemary loading can provide a remarkable effect of stabilization. If the amounts of 0.25 and 0.50 wt% bring small contributions at medium oxidation temperatures, where the oxidation causes the formation of peroxides from the initial polymer (first peak) or from the derivative products (the second peak), the degradation is effectively delayed at higher temperatures exceeding 170 °C (Figure 12a,b). The concentration of 0.75 wt% rosemary extract delivers a significant effect for the preservation of oxidation state in PLA samples. Rosemary ethanolic extract (R) acts as a protective additive against irradiation, as it was found for other systems [22,23], showing a good radical scavenging activity.

The low CL intensities recorded for these last-mentioned samples in comparison with the low-loaded material (Figure 12c) is evidence for the involvement of powder rosemary component in the breaking action on the oxidation chain process. In the non-isothermal CL spectra (Figure 11 and Figure 12), the effect of irradiation on the degradation of PLA, as the main radical source is proven. The presence of polyether will restrain the emission of the PLA intermediates. Consequently, this former peak disappears, and the principal peak presents prominent amplitude. The increase in emission intensities around 200 °C justifies the further degradation of the new configuration.

In Table 4, where the calculated activation energies are listed, the large spectrum of data demonstrates the participation of components to various extents to chemical modifications induced by gamma irradiation, which foresees the radiochemical strength of compounds. The proportion of chitosan is also an important factor that modifies the energetic conditions of oxidative degradation. The comparison between the two composition groups, with and without chitosan, reveals its importance in the degradation. It would be assumed that chitosan is also involved in the building up of the cross-linked configuration, because the values of activation energy are greater as chitosan exists in the irradiated samples, but values are very much decreased with increasing radiation dose. OOT (onset degradation/oxidation temperature) decreases with increasing radiation dose; therefore, degradation will be faster.

### 3.5. Bioactive Compounds Release Study and Migration Kinetics of the Rosemary Extract Components from Irradiated and Non-Irradiated PLA/PEG/CS-Based Films

The migration study was based on the determination of the rosemary active components content in the food simulant, by UV-VIS spectroscopy. For this study, only formulations containing 0.5 wt% R extract were considered.

The registered UV-VIS spectra for the irradiated PLA/PEG and PLA/PEG/CS samples containing the rosemary extract showed an absorption maximum at 275 nm, the same as in our previous investigation, for non-irradiated samples [25], which is shifted by ~10 nm compared with the maximum on the calibration curve, registered at 285 nm [26]. This hypsochromic shift may occur in the absorption spectrum due to chemical interactions (dissociation or reaction with the solvent) and may give information about the intermolecular structural changes and/or perturbation of the electronic states due to some environmental factors [68].

**Figure 13:**
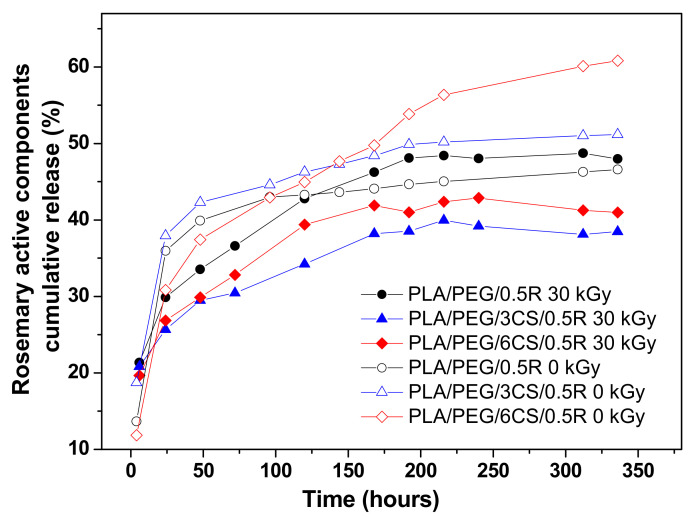
Comparative migration profiles of the bioactive compounds of R from irradiated and non-irradiated PLA/PEG/R and PLA/PEG/CS/R biocomposites.

The rosemary active components migration from the PEG-plasticized PLA/chitosan irradiated films into simulant was studied according to EU regulations (see above), and the results are summarized in Figure 13 and Table 5.

Generally, the migration takes place slower and in a significantly lower quantity for all irradiated samples, compared with non-irradiated ones and especially for CS containing samples [25] (Figure 13).

The release curves from irradiated PLA/PEG/CS-based films prepared by melt mixing, obtained at 40 °C in 50% ethanol solution as food simulant medium, show a quite similar migration behavior for the three compositions tested, with a slower release and lower amounts released, up to a 14-days interval, investigated for the CS-containing samples. The three irradiated compositions studied showed a gradual release in the first 120 h; then after 150 h reached a plateau much later than non-irradiated samples. Sample PLA/PEG/R showed a faster release and the highest amount released, of ~48.7%, while the samples containing CS reached only 38.5% release for the biocomposite film containing 3 wt% CS and 41.2% from the sample containing 6 wt% CS. The migration profiles with a slower cumulative release and smaller released amount at a dose of 30 kGy can be explained by an increase in crosslinking density of PLA and a decrease in material flexibility. This is in accordance with variation in some thermal properties of materials irradiated with such dose. It can be also concluded that these bioactive compounds are active for a longer period in PLA matrix; therefore increasing the shelf-life of packaged products.

In order to have a deeper understanding of the migration mechanism for the active components of the rosemary extract from the irradiated PLA/PEG/CS films, the kinetic parameters were evaluated (Table 5).

The calculated kinetic parameters and the corresponding correlation coefficient values (R^2^) are summarized in Table 5.

The release exponent, *n* values were obtained by fitting the data to a power law model (Equation (1)) [69]:

(1) mtm∞=k·tn

where *m_t_/m_∞_* represents the fraction of bioactive compound(s) released at time *t*, *n* is the release exponent, and *k* is the release rate constant. The *n* < 0.5 values suggest a pseudo-Fickian migration behavior, determined by both swelling and diffusion processes. These data correlate with the release profiles, namely the faster release on the first interval of 120 h may be promoted by the swelling of the film, by ethanol sorption, followed by the second interval, after 150 h from 6 days up to 14 days, with a sustained release behavior determined probably by the interactions that occurred between the components from the film composition. The release rate k constant values demonstrate the slower release from the CS-containing films, with similar values and both lower than that for the PLA/PEG sample (Table 5).

The next evaluation was completed considering the first order kinetic model—Equation (2) [69,70]:

(2) $$ln\left(1-\frac{m_{t}}{m_{\infty }}\right)=-k_{1}\cdot t$$

The obtained results of fitting the experimental data with Equation (2) gave the lower correlation coefficient (R^2^) values; therefore, the first order kinetic model is not the most adequate model to describe the migration process from the studied biocomposites. The *k*_1_ values preserve the same trend, decreasing for CS-containing samples, especially for PLA/PEG/3CS composition.

A simplified migration model was derived from Fick’s second law, was applied for short-term migration and defined as the time for which *m_t_/m_∞_* < 0.6, considering *diffusion* as the main process governing the release of the active components, which occurs from both sides of the film—Equation (3):

(3) $$\frac{m_{t}}{m_{\infty }}=4{\left(\frac{Dt}{\pi l^{2}}\right)}^{1/2}$$

where *D* is the diffusion coefficient and *l* is the film thickness. A plot of *m_t_*/*m*_∞_ versus t^1/2^ should yield a straight line from which the diffusion coefficient can be obtained.

As it can be observed from Table 5, a good fitting was obtained using Equation (3) with correlation coefficient (R2) values of 0.97-0.98, indicating that experimental data are well described by the diffusion model for short-range times, from for the two CS-containing samples, the PLA/PEG/3CS composition has the lowest D value (1.08 × 10^−18^ m^2^/s).

The calculated partition coefficient values, K_p_ (the ratio of the migrant concentration in the film (C_f,∞_) to the migrant concentration in the food simulant system (C_s,∞_)) [25], show that for sample PLA/PEG/0.5R 30 kGy the migrants’ concentration in the film equals the concentration in the food simulant (since K_P_ ~ 1), behavior that correlates well with the percent released, of 48.7%. The K_P_ > 1 values obtained for CS-containing samples show a higher affinity of the migrant for the polymeric film, which can be attributed to the possible interactions between the functional groups and is well correlated with the released percent, below 50% for both samples [39,71].

The overall migration levels obtained are much lower than the overall migration limits imposed for food contact materials, of 60 mg/kg of food simulant, established by the current legislation for food packaging materials in both polar and non-polar simulants [35,36,72]. Also they are significantly smaller than those corresponding to non-irradiated samples. Migration of bioactive components from PEG-plasticized PLA containing R into D1 simulant was slower and with a lower amount released in the case of irradiated samples, especially the CS-containing samples. These results correlate with those obtained by chemiluminescence method. Migration behavior shows a tendency towards Fickian diffusion, with diffusion coefficient values with five orders of magnitude smaller than for non-irradiated samples. 

### 3.6. Antibacterial Activity

Antibacterial activity expressed as the percentage inhibition at 24 h and 37 °C against three bacteria *Salmonella typhymurium*, *Escherichia coli*, and *Stapylococcus aureus* is demonstrated by the data of Table 6 depending on irradiation dose.

For the samples that underwent irradiation treatments, the number of micro-organisms decreased significantly to zero for all biocomposites after gamma irradiation with a dose of 30 kGy.

**Figure 14:**
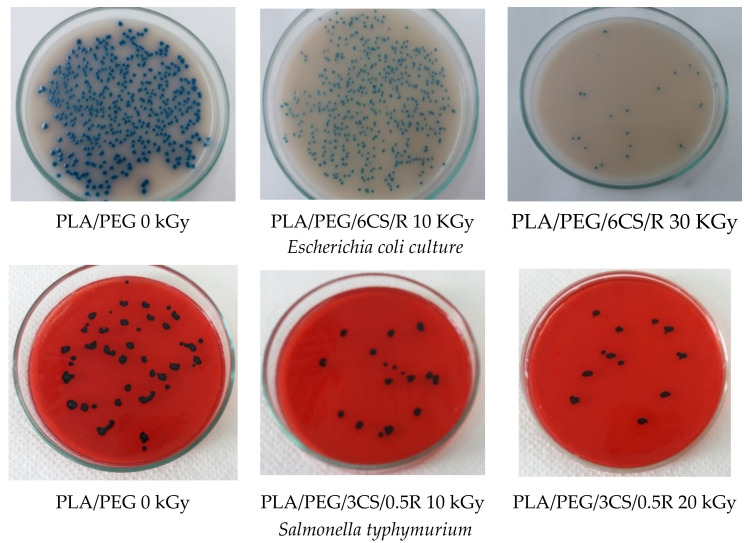

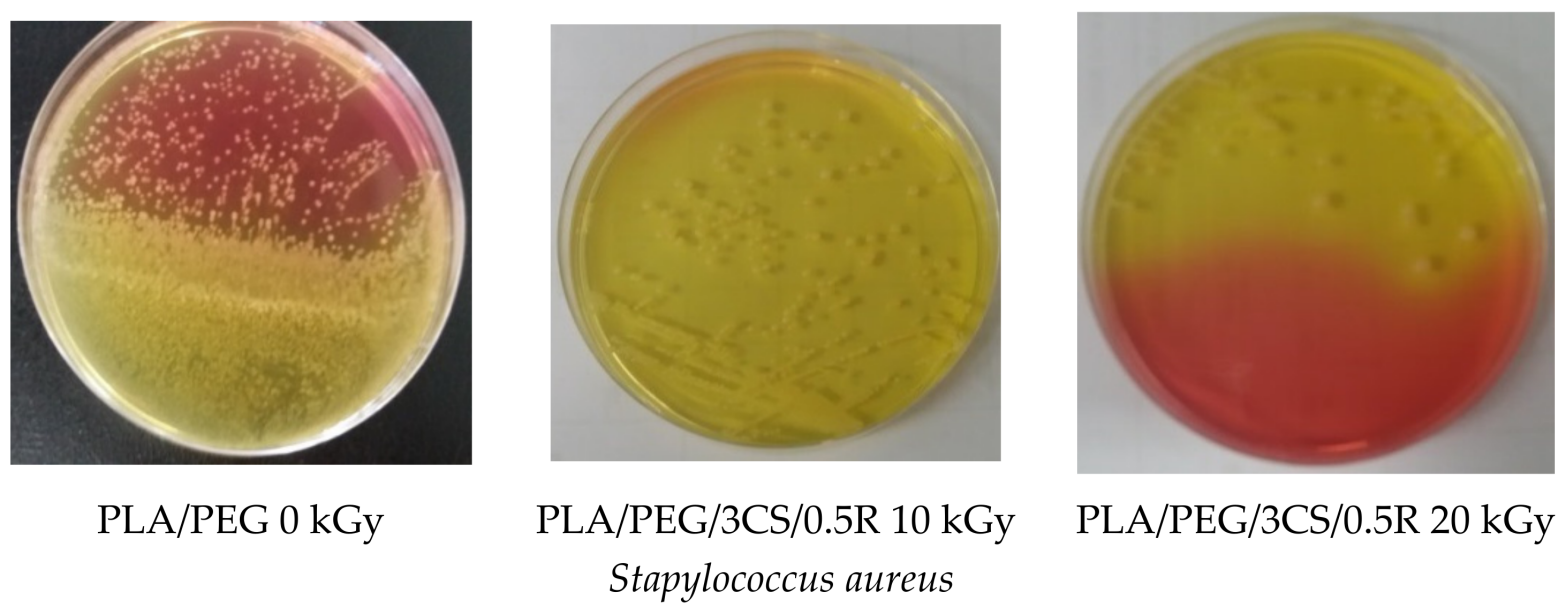
Microscopical aspects of the three bacterial cultures in the presence of PLA-based systems before and after irradiation.

Antimicrobial activity increased with gamma exposure (Figure 14) and because of surface deterioration—see Figure 1 SEM images.

The inhibition after 24 h from seeding in bacterial culture was 100% after gamma irradiation even at a low dose of 10 kGy, as in the case of *Staphylococus aureus* culture. Irradiation destroys much of, or completely, the microbial flora with increased irradiation dose. This happens for PLA/0.75R blend and all biocomposites containing CS and R, which showed a synergetic role to impart both a good antibacterial activity and a protective action against irradiation because of free radical scavenging activity—see chemiluminescence results.

As a general observation, the gamma irradiation destroyed the majority or all of the microbial species.

Matsuhashi and Kume studied the antimicrobial activity of irradiated chitosan (100 kGy under dry conditions) against *Escherichia coli* B/r and they found an effective increase in the activity, and inhibition of the growth of *E coli* completely. The 1 × 10^5^–3 × 10^5^ fraction showed the highest antimicrobial activity [19,. Gamma irradiation (from 10 to 30 kGy) increases antimicrobial activity. At high doses, a decrease in antimicrobial activity of CS is possible because of average molecular weight decrease. Other authors found that the degree of deacetylation of chitin, water binding capacity, fat binding capacity, and antimicrobial activity of the chitosan is improved on irradiation [73].

## 4. Conclusions

The gamma irradiation effects on PLA/(0.25–0.75 wt%)R blends and PLA/(20 wt%)PEG/(3–6 wt%) CS/(0.5%)R biocomposites were studied by SEM, FTIR, DSC, TG/DTG, CL methods, bioactive compounds release and kinetics of migration studies, and antimicrobial activity against *Salmonella typhymurium*, *Escherichia coli*, and *Stapylococcus aureus*. The obtained results showed that the oxidative degradation under gamma irradiation of PLA-based materials is evidenced by modifications in their surface homogeneity, structure, thermal properties, and antimicrobial activity. These modifications are attributed to the morphology evolution of irradiated biocomposites which is affected by the irradiation and presence of natural additives such as chitosan and rosemary extract. It was established that, under a gamma-irradiation treatment with a dose of 10–20 kGy, the PLA-based materials showed minor changes in structure and properties, being suitable for application as packaging and, in addition, after irradiation with such doses their antimicrobial activity against *Escherichia coli*, *Staphylococcus aureus*, and *Salmonella typhimurium* was improved

The presented results are in accordance with those reported by Khalil et al. [74], for gamma-irradiated starch/chitosan/Ag nanocomposites, that the increase in irradiation dose more than 5 kGy leads to a decrease in the composite solution viscosity, the overall crystallinity, and thermal stability. This is because the scission and oxidation of polymer chains increase with the dose of irradiation (from 10 to 30 kGy).

The presence of chitosan in the composition of the materials promotes their degradation. As a result, the studied biocomposites are environmentally degradable materials and gamma irradiation is a promising solution to be applied both for sterilization of such samples when used in the medical field for dose of ~25 kGy and in the treatment of polymer waste at high doses. We expect, based results showing structural changes, and decrease in thermal properties after irradiation, that further irradiation could be a sustainable ecological solution to influence the stability or biodegradation behavior of such materials depending on biocomposites’ composition and irradiation dose. Incorporation of natural components into PLA and further irradiation should be a sustainable economic and ecological solution to confer to materials new characteristics and could accelerate their environmental disintegration after use without negative effects on the environment, being helpful for waste reduction. This will be studied in future research.

## Figures and Tables

**Table 1:** Compositions of the studied PLA-based materials.

Nr.	Sample	Polylactic Acid (PLA) (wt%)	Rosemary Extract (R) (wt%)	Poly(ethylene glycol) (PEG) (wt%)	Chitosan (CS) (wt%)
1	PLA	100	-	-	-
2	PLA/0.25R	99.75	0.25	-	-
3	PLA/0.5R	99.50	0.5	-	-
4	PLA/0.75R	99.25	0.75	-	-
5	PLA/PEG/3CS	77	-	20	3
6	PLA/PEG/3CS/0.5R	76.5	0.5	20	3
7	PLA/PEG/6CS	74	-	20	6
8	PLA/PEG/6CS/0.5R	73.5	0.5	20	6

**Table 2:** Thermal characteristics determined from DSC curves recorded in the 2nd heating run for the non-irradiated and irradiated PLA/R blends and PLA/PEG/CS/R biocomposites.

Sample	Dose (kGy)	T_g_ (°C)	T_cc_ (°C)	T_m_ (°C)
PLA	10	65.7	125.8	150.3; 156.5
	20	65.2	117.1	148.4; 156.2
PLA/0.25R	10	65.7	121.5	157.3
	20	63.7	112.3	158.4
	30	62.4	111.6	148.6; 155.7
PLA/0.5R	10	66.2	124.1	150.6
	20	63.7	121.3	143.4 sh; 156.8
PLA/0.75R	10	64.9	126.5	151.0
	20	63.7	122.3	149.2; 156.3;
	30	63.4	117.9	147.2; 155.5
PLA/PEG/3CS	0	57.6	86.9	148.8
	10	-	82.6	142.5
	20	54.2	83.2	145.1
	30	-	74.6	126.4 sh; 134.9
PLA/PEG/6CS	0	57.2	88.1	149.4
	10	55.4	85.3	136.4 sh; 145.4
	20	54.5	85.4	144.5
	30	-	77.3	136.4
PLA/PEG/3CS/0.5R	0	59.6	89.2	149.6
	10	57.3	85.3	143.6
	20	-	81.7	140.8; 144.5
	30	56.4	84.0	137.6 sh; 144.2
PLA/PEG/6CS/0.5R	0	56.3	89.5	149.4
	10	56.6	84.7	142.8
	20	54.4	83.3	140.5 sh; 145.3
	30	54.0	81.2	140.5

T_g_—glass transition temperature; T_cc_—cold crystallization temperature; T_m_—melting temperatures; sh—shoulder.

**Table 3:** TG data of PLA-based samples irradiated at different doses.

Sample	Dose (kGy)	T_onset_ (°C)	T_10%_ (°C)	T_20%_ (°C)	T_M_ (°C)	T_f_ (°C)	Δw (%)
PLA	10	272.7	304.2	313.5	331.7	356.7	96.4
	20	221	271.6	278.6	302.9	326.4	92.4
PLA/0.25R	10	255.7	304.2	313.5	328.4	358.0	92.5
	20	189.9	248.4	264.9	277.3	317.9	90.7
	30	221.8	267.0	278.8	290.1; 302.9	343.4	89.9
PLA/0.5R	10	227.9	283.5	299.6	320.5	339.2	94.5
	20	189.5	245.9	262.3	279.4	313.4	94.5
PLA/0.75R	10	232.3	274.5	292.8	298.2	319.7	94.5
	20	211.2	257.9	271.6	292.5	319.9	94.5
	30	234.6	274.2	289.2	303.1	337.1	97.1
PLA/PEG/3CS	0	203	276.7	301.6	351.5; 401.2	425	98.6
	10	163	205.2	270.4	322.5; 398.7	425	98.6
	20	163	237	280.06	317.9; 397.9	425	98.6
	30	140	213.8	281	325.0; 399.1	427	97.8
PLA/PEG/6CS	0	217	277.2	300.8	360; 402	439	98.8
	10	217	258.6	289.1	314.9; 397.5	439	97.9
	20	215	258.6	278.7	322.7; 399.7	439	97.3
	30	214	251.9	280.6	311.9; 399.7	437	97.7
PLA/PEG/3CS/0.5R	0	166	283	285	355.3; 403.2	437	98.5
	10	136	255	287	329.7; 397.7	438	98.8
	20	136	251	282	319.7; 398.6	438	98.8
	30	125	255	277	314.5; 395.3	430	98.6
PLA/PEG/6CS/0.5R	0	166	282	315.2	359.2; 403	510	97.8
	10	139	245	293	330.7; 399.7	486	96.5
	20	139	245	288.9	326; 397.9	492	96.4
	30	139	245	293.9	323.1; 398.8	486	95.4

T_onset_—the onset temperature, at which the thermal degradation starts; T_10%_,T_20%_—temperature corresponding to the 10% and 20% mass loss; T_M_—temperature corresponding to the maximum rate of mass loss; T_f_—temperature corresponding to the end of the decomposition; ΔW—residual mass at 500 °C.

**Table 4:** Onset degradation/oxidation temperature (OOT) and activation energies of irradiated biocomposites.

Sample	Dose (kGy)	OOT (°C)				Correlation Factor	Activation Energy (kJ/mol)
		Heating Rate (°C/min)					
		3.7	5.0	10.0	15.0		
Former peak							
PLA	10	85	88	100	115	0.97355	46.72
	20	83	85	95	111	0.95152	48.38
	30	81	86	93	108	0.95576	52.79
PLA/0.5R	10	85	87	98	110	0.97662	55.79
	20	83	86	96	110	0.98547	51.78
	30	81	85	96	110	0.97925	52.80
Major peak							
PLA	10	188	205	220	228	0.96331	59.36
	20	180	194	208	221	0.98400	58.36
	30	173	189	201	215	0.97138	55.45
PLA/0.5R	10	189	202	210	222	0.96548	76.74
	20	185	197	206	220	0.97323	71.50
	30	179	190	201	215	0.90371	67.26
PLA/PEG/3CS	10	207	215	230	240	0.99902	74.91
	20	198	206	225	232	0.99463	70.92
	30	189	200	212	230	0.98046	62.77
PLA/PEG/3CS/0.5R	10	205	215	225	232	0.98261	98.85
	20	199	208	220	228	0.99292	89.21
	30	189	205	212	218	0.91934	81.39
PLA/PEG/6CS	10	189	205	212	216	0.90206	84.13
	20	185	202	208	215	0.90953	77.32
	30	181	195	205	215	0.96790	70.91
PLA/PEG/6CS/0.5R	10	208	217	231	238	0.99313	87.63
	20	203	214	228	232	0.97303	85.63
	30	201	210	222	234	0.99275	80.15

**Table 5:** Kinetic parameters of the migration of the active components of the rosemary extract into simulant D1 from plasticized PLA blends and biocomposites.

Samples	Peppas/Power Law Model				First Order Kinetic Model		Diffusion Model		K_P_	C (mg/kg)
	n	R^2^	k × 10^−3^ (h^−n^)	R^2^	k_1_ (h^−n^)	R^2^	D (m^2^/s)	R^2^		
PLA/PEG/0.5R	0.37	0.99	85.12	0.98	5.20 × 10^−3^	0.84	1.7 × 10^−13^	0.98	1.06	19.2
PLA/PEG/3CS/0.5R	0.23	0.98	147.78	0.99	5.17 × 10^−3^	0.81	2.05 × 10^−13^	0.97	0.95	14.1
PLA/PEG/6CS/0.5R	0.38	0.99	72.92	0.99	3.7 × 10^−3^	0.92	1.05 × 10^−13^	0.99	0.64	18.4
PLA/PEG/0.5R 30 kGy	0.26	0.99	7.46	0.99	12.52 × 10^−5^	0.87	1.78 × 10^−18^	0.97	1.08	6.67
PLA/PEG/3CS/0.5R 30 kGy	0.19	0.99	6.54	0.99	8.83 × 10^−5^	0.91	1.08 × 10^−18^	0.98	1.6	5.8
PLA/PEG/6CS/0.5R 30 kGy	0.21	0.99	6.94	0.99	11.46 × 10^−5^	0.88	1.23 × 10^−18^	0.98	1.44	5.84

**Table 6:** Antibacterial activity as the percentage inhibition of the non-irradiated and irradiated PLA-based blend biocomposites against *Salmonella typhymurium*, *Escherichia coli*, and *Stapylococcus aureus*.

Sample	*Salmonella typhymurium*		*Escherichia coli*		*Stapylococcus aureus*		
	0 KGy	30 KGy	0 KGy	30 KGy	0	10 KGy	30 KGy
PLA	32	100	53	100	43	100	100
PLA/0.25R	52		61		39	100	
PLA/0.5R	52	100	71	100	87	31	100
PLA/0.75R	55		94		42	100	
PLA/PEG	29	100	69	100	44	58	100
PLA/PEG/3CS	58		73		34	100	
PLA/PEG/6CS	58		73		22	100	
PLA/PEG/0.5R	48	100	76	100	88	100	100
PLA/PEG/3CS/0.5R	81	100	76	100	92	78	
PLA/PEG/6CS/0.5R	90	100	82	100	86	56	100

## Data Availability

Not applicable.
